# Determinants of Use of Insecticide Treated Bednets Among Caregivers of Under Five Children in an Urban Local Government Area of Osun State, South-Western Nigeria

**DOI:** 10.5539/gjhs.v7n2p20

**Published:** 2014-09-25

**Authors:** O. A. Esimai, O. O. Aluko

**Affiliations:** 1 Department of Community Health, Obafemi Awolowo University, Ile Ife, Nigeria

**Keywords:** insecticide treated nets, under five caregivers, awareness, ownership, use

## Abstract

In Sub Sahara Africa, the use of Insecticide Treated Nets (ITNs) is one of many strategies of Roll Back Malaria (RBM) initiatives to reduce malaria burden. This study therefore assessed the current use of insecticide treated nets and the determinants of its use among the caregivers of under five children in an urban local government area in Osun state, Nigeria. The study utilised a cross-sectional design among caregivers of under-five children selected from households by multistage sampling technique. The study collected quantitative data using pretested semi structured, interviewer administered questionnaire while factors that determine the current use of ITN were identified using multi linear logistic regression.

The study revealed that 54.4% caregivers of under-five children were aware of ITNs as one of the malaria preventive measures, 49.1% had good knowledge of ITN and 38% agreed with the use of ITNs. Thirty four percent had access to ITNs, 32.3% owned at least one ITN with 30.3% reported been given free in the health care facilities. Thirty three percent had ever used and the foremost reasons for non-use are not readily available and expensive. Only 18.5% currently used ITNs and challenges faced were not easy to treat, difficult to set up and no place to keep it. Marital status, knowledge of ITN, attitude towards ITN, ownership of ITN and free ITN were factors that determined the use of ITNs amongst the respondents.

There is a need to ensure intensive awareness on ITNs through campaigns and embark on its mass distribution to the public to enhance use.

## 1. Introduction

Malaria burden in Sub Saharan African (SSA) Countries has declined substantially with multi sectoral programme support to vulnerable population (O’Meara, Mangeni, Steketee, & Greenwood, 2010). Despite this, about 60% of malaria cases worldwide and over 80% of malaria mortality occur in occur in SSA. It has also been reported that a child under five dies from malaria every 30 seconds and children who survive severe malaria may suffer irreversible functional disability for the rest of their lives (Global Health, 2009). Nigeria has about 25 percent of the malarial disease burden in Africa, contributing significantly to the one million lives lost per year in the region, mostly amongst children and pregnant women. Malaria related deaths account for about 11 percent of maternal mortality, 25 percent of infant mortality, 30 percent of under five mortality resulting yearly in about 300,000 deaths (National Planning Commission/National Malaria Control Programme/ICF International [NPC/NMCP/ICF], 2012). In fact, severe malaria, from a hospital based study in Nigeria revealed the proportion of those admitted for severe malaria during the study period was 13% (O’Meara et al., 2010).

The global commitment of Roll Back Malaria (RBM) programme is to reduce by 50%, the malaria disease and associated death by 2010, a target set by leaders of 44 African nations in Abuja, Nigeria in April 2000. In addition, the specific targets of malaria control under the RBM initiative are: the use of ITN by at least 60% of people at risk of malaria (young children and pregnant women) and access to effective preventive treatment by at least 60% of pregnant women (FMOH, 2000; WHO, 2008). Household ownership and use of Insecticide Treated Nets (ITNs) is one of the main strategies of Roll Back Malaria (RBM) programme with a view to reducing malaria burden. In this regard, routine and continuous use of ITNs can reduce malaria transmission by up to 90% and prevent up to 44% of all cause mortality among under five children (Fragan, Noor, Akinwale, Cousa, & Snow, 2007). The government of Nigeria with the support of development partners distributed 30 million ITNs and plans to further distribute additional 30 million ITNs to achieve universal net coverage (one ITN for every two people) in the country (NPC/NMCP/ICF, 2012).

Forty two percent owns at least one ITN, 23% of the households uses ITN and 29% of children under five years old in all the households slept under an ITN, the night before the survey (NPC/NMCP/ICF, 2012). Many studies revealed that multiple factors determine the ownership and use of ITNs. Therefore, cost among others, has been identified as a major determinant for non-ownership of ITN (Khamlome et al., 2007; Noor, Amin, Akhwale, & Snow, 2007). In addition, the relative scarcity at local vendors is another deterrent of ITN ownership and subsequent use across Africa (Pettifor, Taylor, Nku, Duvall, & Tabala, 2008). Other factors that affect ownership and / or use of ITN were the house construction design, household sleeping facilities and sleeping arrangements especially in large family and competing needs of the household members in the context of the family and the communities (Miller, Korenromp, Nahlen, & Steketee, 2007). Sleeping space and sleeping patterns also determine the possibility of hanging and sleeping under ITN (Dash & Yadav, 2008).

The factors that played a role in determining use of ITNs in previous studies in Edo and Kwara states in Nigeria were non-availability of the ITNs at close distances from their domain, level of education, marital status, lack of awareness and high cost price of ITN (Wagbatsoma et al., 2010; Musa et al., 2009). A study in Kenya reported factors such as knowledge of ITN, level of education, marital status and occupation of caregiver affected ITN ownership and use by under five children in households. A recent review on community acceptance of bed nets revealed that various factors influences the use of bed nets, including cultural, behavioural and demographic factors, ethnicity, accessibility, gender relations and seasonality of Malaria (Heggenhougen, Hackenthal, & Vivek, 2003). Free distribution of ITNs, otherwise called ‘catch-up’ followed by routine provision of ITNs or using subsidy through vouchers for ITNs to vulnerable population in public health clinics or commercial outlets (keep-up) has dramatically increased national coverage and use in the intervention communities (Grabowsky, Nobiya, & Selanikio, 2007).

The current study therefore assesses the determinants of ITNs use among caregivers of under five children to drive evidence-based intervention strategies to neutralise negative and/or reinforce positive factors that emerged through this study in the study area.

## 2. Methodology

The study was a cross sectional descriptive survey of care givers of under five children in Ife Central Local Government Area (LGA) of Osun State, Nigeria. Two hundred care givers were selected by multistage sampling technique; the first stage involved the selection of 3 wards from 11 wards in the LGA using simple random sampling technique. Thirteen streets were selected from an average of 62 streets by simple random sampling technique, the first house in the selected streets was determined by spinning a bottle at the centre of the street and subsequent houses were selected by moving clockwise. The eligible households were recruited from the houses. The information on knowledge of ITN, attitude towards ITN, access, ownership and use of ITN were collected using semi structured interviewer administered questionnaire.

The data was analysed using Stata software version 12.0. The attitude was rated using 3 likert scale- agree, disagree and indifferent. Attitude classified into positive and negative attitude based on computation of the responses rated in 3 likert scale. Knowledge was classified as good, fair and poor based on categorisation of responses to five questions i.e 4-5 was classified good, 2-3 as fair and less than 2 as poor. Data was analysed using descriptive and inferential statistics and presented in frequency distributions and subjected to multi linear logistic regression to ascertain determinants of use of ITN by care givers. The study was approved by the ethical and research committee of Obafemi Awolowo University Teaching Hospital, lle-lfe.

## 3. Results

Of the 200 caregivers recruited, only 195 consented to be interviewed giving a response rate of 97.5%.

The mean age of the respondents was between 19 and 60 years. All the caregivers are females, 88.7% are married, 56.3% had primary level education and 63% are unskilled. Most (79%) are Christians (Table 1).

**Table 1:** Socio-demographic characteristics of care givers of under five children (N=195)

Variable	Frequency	%
Age (Years)		
19	10	5.1
20-29	87	49.6
30-39	78	40.0
>40	20	10.3
Marital status		
Single	18	9.2
Married	173	88.7
Others	4	2.0
Level of Education		
No formal education	16	8.2
Primary	100	56.3
Secondary	65	33.3
Tertiary	14	7.2
Occupation		
Unskilled	123	63.0
Skilled	35	18.0
Professional	37	19.0
Religion		
Christian	154	79.0
Muslim	38	19.5
Others	3	1.5

Table 2 revealed that more than half respondents (54.6%) are aware of ITNs as a method of malaria prevention, about half of them had good knowledge of ITN and slightly above a third had positive attitude towards ITN. The respondents received their information on ITN from media (87.2%) and hospital (61.5%).

**Table 2:** Awareness, knowledge of and attitude of care givers towards ITN

Awareness (n=195)	Frequency	%
Heard of ITN	106	54.4
Never heard	89	45.6
Knowledge (n=106)		
Good	52	49.1
Fair	43	40.6
Poor	11	10.3
Attitude (n=195)		
Positive Negative	74 121	38.0 62.0

**Table 3:** Logistic regression on determinants of ITN use by caregivers of under five children

Variables	Odds ratio	95% CI	P value
Age of caregivers	0.51	0.14-1.86	0.31
Level of Education	1.30	0.52-3.21	0.56
Occupation	0.78	0.57-1.07	0.12
Marital status	4.63	1.58- 13.5	0.005*
Knowledge	0.21	0.07-0.63	0.005*
Attitude	11.2	2.18-56.9	0.004*
Own ITN	0.05	0.01-0.18	0.0001*
Free ITN	8.26	2.34-29.2	0.001*
Access to ITN	1.24	0.20-7.62	0.82

**Figure 1:**
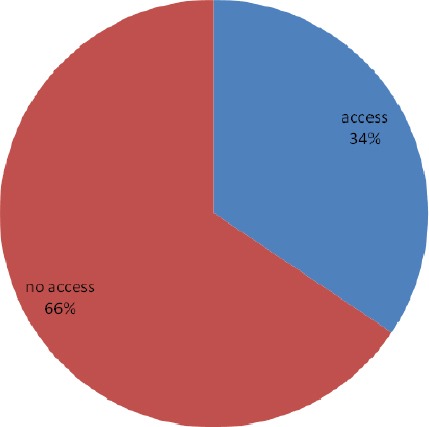
Access to insecticide nets

**Figure 2:**
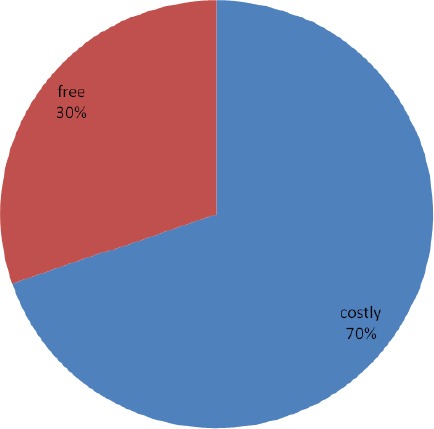
Cosr of Insecticide Treated Nets

**Figure 3:**
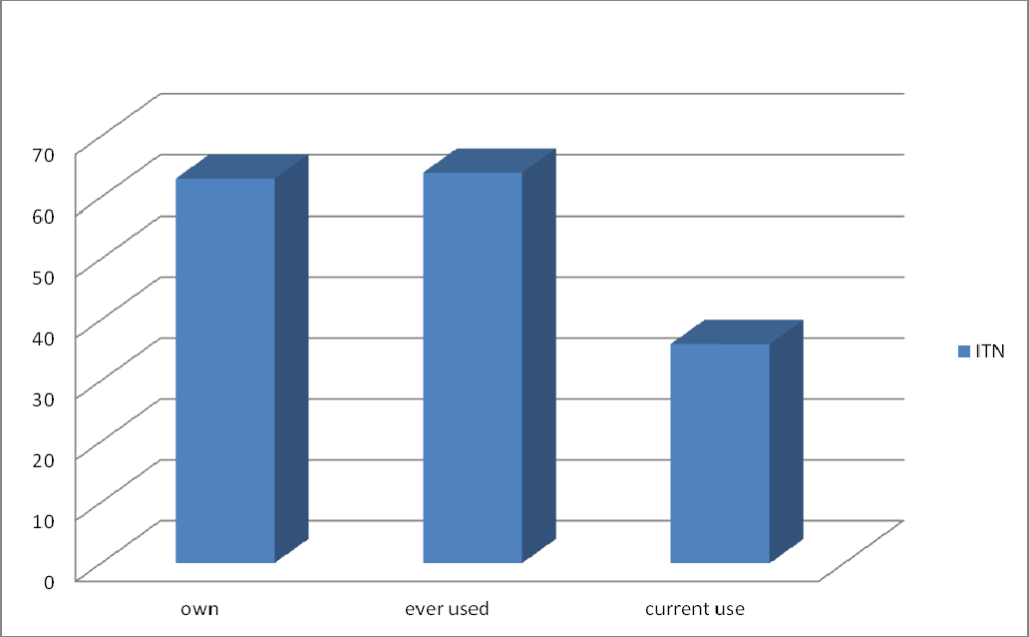
Ownship and use of ITN

Slightly above a third (34.4%) had access to ITN, less than a third (30.3%) got it free (Figures 1 & 2). Thirty two percent owned ITN, about a third (32.8%) had ever used ITN and less than one fifth (18.5%) are currently using ITN (Figure 3).

The reasons giving by 67.2% who had never used ITN include “it was not readily available (13.0%) and expensive (13.7%)”. Some of the challenges experienced by respondents currently using ITN are “it was not easy to treat (0.1%), it is difficult to set up (0.1%) and there is no space to keep it (0.2%)”.

The respondents’ age, level of education, occupation and access to ITN had no significant influence on the use of ITN (p>0.05). Marital status, knowledge of ITN, attitude towards ITN, ownership of ITN and free ITN were factors that determined the current use of ITN by the care givers. The odds of married caregivers who used ITN are 5 times more than the odds of single or unmarried caregivers. Similarly the odds of caregivers with positive attitude who used ITN was 11 times more than the odds of caregivers with negative attitude and the odds of caregivers who got ITN free, used ITN 8 times more than the odds of caregivers who had to pay for it. However, the caregivers with no or poor knowledge and did not own ITN were not likely to use ITN.

## 4. Discussion

The control of malaria across the divergent spectrum of transmission requires good knowledge and awareness about the aetiology and appropriate preventive measures among the resident community, within the existing belief and cultural practices to ensure positive health seeking behaviour.

The level of awareness in the present study was lower than the high level of awareness (88.5%) on ITN in a study in Kenya (Malusha, Mwanzo, Yitambe, & Mbugi, 2009). This might be due to differences in study locations and large sample size used. However the level of awareness was higher than 36% reported among pregnant women attending primary health care facilities in Kwara state, North central Nigeria (Musa, Saludeen, & Jimoh, 2009). The knowledge of caregivers on ITN was about half, this was higher than the report of low knowledge in a study carried out among pregnant women in PHC facilities in Kwara state (Musa, Saludeen, & Jimoh, 2009). However, lower than 85.2% reported in a study carried out amongst pregnant women attending antenatal clinic in University of llorin Teaching Hospital (Omokanye et al., 2012). The differences might be the study population and use of health facilities). Similarly, the finding was higher than the report of 16 % in Malaria Indicator Survey (NPC/NMCP/ICF International, 2012).

The proportion of caregivers who owned ITN at the time of survey was about a third and they got it free, mostly from the health care facilities. This was far lower than the report of 62.4% of ownership of at least one net in a study in Eastern Ethiopia. But the finding of about a third having net free from the hospital was higher than the report of 15.3% of households in Eastern Ethiopia getting free ITN from local health center (Sibhatu, Ayalu, & Haji, 2012). The finding of the present study of about a third in possession of at least one ITN was lower than the report of 58% households in possession of at least one ITN in a household survey in Rubodu ward in Kuje local government Area, Abuja (Ashikeni, Envuladu, & Zoakah, 2013). The high proportion of household ownership of ITN nets in previous studies might be due to presence of national malaria control programme where respondents are provided with ITN. Similarly, the finding of about a third in possession of ITN was below 69.2% having treated nets in a study carried among caregivers in Eastern Kenya (Malusha, Mwanzo, Yitambe, & Mbugi, 2009). The finding of the present study was also below the report of 42% households having at least one ITN in malaria indicator survey 2010 (NPC/ NMCP/ICF, 2012). Over a third of the care givers in this study had access to ITN this was far below the finding of half of the nets available in the household been accessible to under fives in a study carried out amongst caregivers in Eastern province in Kenya (Malusha, Mwanzo, Yitambe, & Mbugi, 2009). In addition, the present study revealed that less than a third of the caregivers ever used ITN. This finding was consistent with the report of less than a third of pregnant women attending PHC centres in Kwara state using ITN (Omokanye et al., 2012). The proportion of caregivers currently using ITN (18.5%) was similar to 19% of pregnant mothers who reported current use of ITN in kwara state (Musa, Saludeen, & Jimoh, 2009). Similarly the finding of the present study on current use was similar to 21.5% reported in a study in Eastern Ethiopia (Sibhatu, Ayalu, & Haji, 2012).

The determinants of use of ITN by caregivers showed that marital status, knowledge on ITN, attitude towards ITN, ownership of ITN and free distribution of ITN were positively associated with its use by care givers of under five children. Caregivers who had low knowledge of ITN are less likely to use it while married women are four times more likely to use ITN. These findings are supported by Malusha, Mwanzo, Yitambe, and Mbugi (2009) where the knowledge of ITN and marital status were positively associated with use of ITN in a study among caregivers in Eastern Kenya. Similarly the finding of the present study was supported by the report of women’s knowledge of ITNs as a significant factor associated with ITN use (Sibhatu, Ayalu, & Haji, 2012). The report of significant association between increasing level of education and use of ITN (Oresanya, Hoshen, & Sofola, 2008) was contrary to the non significant association observed in the present study. There was a positive association between attitude of caregivers to ITN and use of ITN. This was in agreement with previous report of a review of community acceptance of bed nets where behavioural factors were among those identified (Heggenhougen, Hackenthal, & Vivek, 2003). The finding of the present study was supported by the report that age of respondent and educational level had no significant influence on ITN use (Musa et al., 2009). The report of age, educational status, marital status as factors not associated with net use (Sangare et al., 2012) was consistent with the present finding of no significant association between age and educational status and use of ITNs. The finding of a significant association between marital status and use of ITN was supported by the report of marital status as one of the factors that determined use for ITNs in a study carried out in Edo state (Wagbatsoma & Aigbe, 2010). Similarly, the significant association between marital status and use of ITN was supported by the report of marital status having significant association with utilisation of ITN in a study conducted among pregnant women in attending antenatal clinic in University of llorin Teaching Hospital (Omokanye et al., 2012). The finding of the present study on ownership of ITN as a determinant was in agreement with the findings of Sangare et al. (2012) where having more than one net was significantly associated with the likelihood of sleeping under a net at all times during pregnancy. Free distribution of ITNs (catch-up) followed by provision of subsidized ITNs vouchers for the vulnerable people in the society through health care centres or commercial outlets (keep-up) has significantly increased coverage and usage in the intervention areas (Grabowsky, Nobiya, & Selanikio, 2007). This report was in agreement with the finding of a significant association between free distribution of ITN and use of ITN in the present study.

## 5. Conclusion

The use of ITN by caregiver of under five children was low despite the level of awareness and knowledge which are on the average. The low level of ownership of ITN might explain the low current use of ITN among caregivers. The proportion of caregivers who had access to ITN reported it was gotten from the hospital during attendance for other services. Factors such as knowledge of ITN, attitude towards ITN, marital status, ownership of ITN and free ITN were determinants of use of ITN by under five caregivers.

There is a need to continually educate the caregivers on the importance of using ITN routinely and consistently. There is a need for intervention programme that will anchor large scale distribution of ITNs in the communities to increase accessibility and availability. This will in turn improve ownership and on the long run use of ITN.

## Limitation

The responses were self reported and the caregivers were not followed up to check if they actually own or use the ITN.
